# Supplementary material for: Genetic removal of Nlrp3 protects against age-related and R345W Efemp1-induced basal laminar deposit formation
Source: Cell Death Dis. 2025 Nov 6;16(1):803. doi: 10.1038/s41419-025-08104-y (PMC12592729; doi:10.1038/s41419-025-08104-y)
Supplement: Supplementary file 1 — Supplemental Methods and Legends [file 41419_2025_8104_MOESM1_ESM.docx]

Genetic removal of Nlrp3 protects against sporadic and R345W Efemp1-induced basal laminar deposit formation

Antonio J. Ortega^1*^, Steffi Daniel^1*^, Marian Renwick^2^, Pravallika Kambhampati^1^, Krista N. Thompson^2^, Gracen E. Collier^2^, Emily L. Baker^1^, Hasan Zaki^3^, and John D. Hulleman^1, Ω^

^1^ Department of Ophthalmology and Visual Neurosciences, University of Minnesota, 2001 6^th^ St. SE, Minneapolis, Minnesota, 55455, United States

^2^ Department of Ophthalmology, University of Texas Southwestern Medical Center, 5323 Harry Hines Blvd, Dallas, Texas, 75390, United States

^3^ Department of Pathology, University of Texas Southwestern Medical Center, 5323 Harry Hines Blvd, Dallas, Texas, 75390, United States

* These authors contributed equally to this work.

^Ω^Tel.: 1 612 624 8267

^Ω^Email: [hulleman@umn.edu](mailto:hulleman@umn.edu)

**SUPPLEMENTAL MATERIALS AND METHODS**

Conscious intraocular pressure (IOP) measurement. Mice were gently restrained by first placing them in a soft, clear plastic cone (DecapiCone, Braintree Scientific, Braintree, MA) and then securing them in a custom-made restrainer. This restrainer was positioned on a height-adjustable platform. After allowing a few minutes for acclimation, conscious IOPs were measured during the daytime using a TonoLab rebound tonometer (Colonial Medical Supply, Franconia, NH).

Slit-lamp. Slit-lamp imaging was performed on anesthetized mice (2.5% isoflurane and 0.8 L/min oxygen). Anterior chamber phenotypes were evaluated using a slit lamp (Haag-Streit Diagnostics, Mason, OH) and recorded with a digital camera (Canon EOS Rebel T6 DSLR, 18.0-megapixel CMOS image sensor and DIGIC 4+ Image Processor). All images were captured using consistent camera settings for brightness and contrast, with the "auto-focus" option enabled.

Body composition analysis. Differences in mice body composition were assessed using a Bruker MiniSpec mq20 (Bruker Optics, Billerica, MA). Compositional changes in total body weight, body fat weight, lean tissue weight, fluid weight, percent weight of fat, and percent weight of lean tissue were assessed. Mice were scanned twice per session and the values were then averaged and subjected to comparison across genotypes using a two-way ANOVA and Tukey’s multiple comparison test. n = > 3.

Bone marrow-derived macrophage (BMDM) isolation and treatment. To validate Nlrp3 inflammasome pathway inactivation (i.e., in mice lacking Nlrp3 or Casp1), we tested the release of the proinflammatory cytokine, IL-1β from BMDM cells originating from mice of each genotype. Femoral and tibial bone marrow from 2-month-old mice from each cohort were collected, cultured, and differentiated for 6 d into BMDMs using Iscove's Modified Dulbecco's Medium (IMDM) medium (ThermoFisher, Waltham, MA) supplemented with 10% FBS (Omega Scientific, Tarzana, CA), 25 ng/mL macrophage colony stimulating factor (M-CSF, ThermoFisher), 1% nonessential amino acids (ThermoFisher), and 1% penicillin-streptomycin (ThermoFisher). Cells were then replated onto a 12 well plate at 1.2 x 10^6^ cells/well and cultured overnight (Corning, Corning, NY). Cells were then subject to one of four treatments, 3 h 500 ng/µL lipopolysaccharide (LPS, Sigma, St. Louis, MO), 1 h 10 µM nigericin (Cayman, Ann Arbor, MI), a combination of LPS and nigericin, or untreated. IL-1β release into the cell culture media was assayed using a Lumit Mouse IL-1β Immunoassay (Promega, Madison, WI) followed by reading on a GloMax plate reader (Promega).

Microglia Soma Size Analysis. The same image stacks used for microglial cell density analysis (Figures 6 and 9) were also used to assess microglia soma size. Analysis was performed using the Microglia Morphology Analysis ImageJ Plugin, which is publicly available on GitHub (https://github.com/ciernialab/MicrogliaMorphology). Briefly, the plugin first applies a quality filter to each Z-stack by selecting the sharpest, least motion-distorted frames across planes to generate a cleaned stack, which is then verified by the user. These stacks are subdivided into substacks and projection-averaged. An automated soma detector identifies cell bodies and records their X–Y coordinates. Binary masks are generated for each soma, and an algorithm traces provisional soma outlines for quality control. The final approved contours are used to extract soma area, perimeter, and centroid for morphometric analysis.

Cytokine Profiling Array. To gain further insight into inflammation-related pathways potentially involved in BLamD formation, we took an unbiased cytokine/chemokine profiling approach. Twelve-month-old WT, R345W^+/+^, Casp1^-/-^ R345W^+/+^, Nlrp3^-/-^ R345W^+/+^ mice were deeply anesthetized using an i.p. ketamine/xylazine injection. Once unresponsive to foot pinch, the mice were perfused with 20 mL of heparinized saline to fully clear blood from the choroid. Eyes were immediately enucleated and dissected by cutting along the ora serrata to remove the anterior portion of the eye and the lens. The neural retina (NR) and retinal pigmented epithelium (RPE)/eye cups were then carefully separated, homogenized in PBS with protease inhibitors (ThermoFisherScientific) and 1% triton X-100 (Sigma-Aldrich) and frozen overnight at -80°C. Thawed samples were centrifuged and total sample protein was normalized across samples to 100 µg for NR and 200 µg for RPE/eyecups. Samples were then assayed using the Mouse XL Cytokine Array Kit ARY028 (R&D Systems, Minneapolis, MN) and imaged by following the Proteome Profiler Arrays with LI-COR Detection protocol (R&D Systems). The cytokine levels were quantified using the Protein Array Analyzer for ImageJ add-on by Gilles Carpentier (<http://image.bio.methods.free.fr/ImageJ/?Protein-Array-Analyzer-for-ImageJ>).

**SUPPLEMENTAL FIGURE LEGENDS**

**Sup. Fig. 1.** RPE subpopulation morphometry is similar in WT and R345W^+/+^ mice. (A-D) Graphs comparing cell area (A), aspect ratio (B), hexagonality (C), and neighbors (D) for the three RPE subpopulations in WT and R345W^+/+^ at 22 mo. To assess the statistical significance of the observed differences in morphometric features among the subpopulations, a two-way ANOVA was performed. Mean ± S.D., n = 6 for WT, n = 7 for R345W^+/+^.

**Sup. Fig. 2.** Corneal opacity, IOP, and RGC density are similar between WT and R345W^+/+^ mice. (A) Unremarkable representative slit lamp images from WT and R345W^+/+^ mice (14 mo, n = 5-6 mice). (B) IOP measurements in WT and R345W^+/+^ mice (14 mo, n = 5-6 mice; 10-12 eyes) are not statistically different. Unpaired t-test. Mean ± S.D. (C) RGC density in WT and R345W^+/+^ as determined by RBPMS staining (17 mo, n = 7-8 eyes) is also not different between genotypes. DAPI (blue) was used as nuclear stain (Scale bar = 100 µm). (D) Graph representing RGC cell densities that remain unchanged in R345W^+/+^ mice compared to WT. Unpaired t-test. Mean ± S.D.

**Sup. Fig. 3.** Serum cholesterol, triglycerides, and body composition are not different in WT and mutant F3 mice. (A) cholesterol or (B) triglyceride analysis was performed from serum obtained from 16 mo mice, n = 2-5, mean ± S.E.M. (C) Body composition was evaluated by a micro-CT scanner (Bruker) at 12 mo (n = 6-10 individual mice). Only statistical differences in body composition were observed in the mass of male vs. female mice. One-way ANOVA. * p < 0.05, ** p < 0.01.

**Sup. Fig. 4.** Re-presentation of data from Fig. 2 by genotype and BLamD size/coverage. (A-D) to obtain a better longitudinal view of BLamD size and coverage (growth), data from Fig. 2 was re-plotted according to genotype and across ages. Two-way ANOVA with Kruskal-Wallis test for multiple comparisons. **** p < 0.0001. n = 6 mice/genotype and age group (3 male, 3 female) except for 20 mo mice (n = 3 females/genotype). Median along with 1^st^ and 3^rd^ quartiles plotted.

**Sup. Fig. 5.** Confirmation of functional impairment of the Nlrp3 inflammasome using BMDM. (A-D) An IL1β Lumit assay was used to evaluate cytokine production in differentiated BMDM isolated from 2 mo mice after treatment with LPS and/or nigericin. 8 mice in total used, all female. One mouse for each genotype. 6 replicate wells for each treatment group and the experiment was repeated twice and data combined.

**Sup. Fig. 6.** Soma size calculation across different retinal planes. WT (20 mo), R345W^+/+^ (20 mo), and Nlrp3^-/-^ R345W^+/+^ (22 mo) mice are compared here for soma size differences of Iba1+ (green) stained microglia in the ganglion cell layer (GCL), inner plexiform layer (IPL), and outer plexiform layer (OPL). Graphs representing soma sizes in each of the specified layers showing significant increase in the IPL and OPL layers (WT vs R345W^+/+^) and significant decrease in microglia sizes in the IPL and OPL layers (R345W^+/+^ vs Nlrp3^-/-^ R345W^+/+^). * p < 0.05, ** p < 0.01, *** p < 0.001. Mean ± S.D.

**Sup. Fig. 7.** Re-presentation of data from Fig. 6 and Fig. 9 in totality. Since data from these two figures were analyzed together, we present the data from Fig. 6 and from Fig. 9 together for comparison. Two-way ANOVA. * p < 0.05, ** p < 0.01, *** p < 0.001, **** p < 0.0001. Mean ± S.D.

**Sup. Fig. 8.** Cytokine array analysis of RPE/choroid and neural retinal (NR) tissues in WT, R345W^+/+^, and Nlrp3^-/-^ R345W^+/+^ mice. (A-D) Samples were collected from perfused 12 mo WT, R345W^+/+^, and Nlrp3^-/-^ R345W^+/+^ female mice. In total, 111 cytokines were assayed in duplicate for each tissue sample from all genotypes. The top 11 cytokines in the RPE/choroid (A) and top 8 cytokines measured in the NR (B) with the greatest fold change in expression were highlighted. These same cytokines were all downregulated in RPE/choroid (C) and NR (D) in Nlrp3^-/-^ R345W^+/+^ samples, reverting their abundance to near WT-levels in many cases. Cytokines spotted in duplicates. One mouse per genotype. 8 mice total used for these arrays.
