# Supplementary figures and images for: Genetic removal of Nlrp3 protects against age-related and R345W Efemp1-induced basal laminar deposit formation
Source: Cell Death Dis. 2025 Nov 6;16(1):803. doi: 10.1038/s41419-025-08104-y (PMC12592729; doi:10.1038/s41419-025-08104-y)

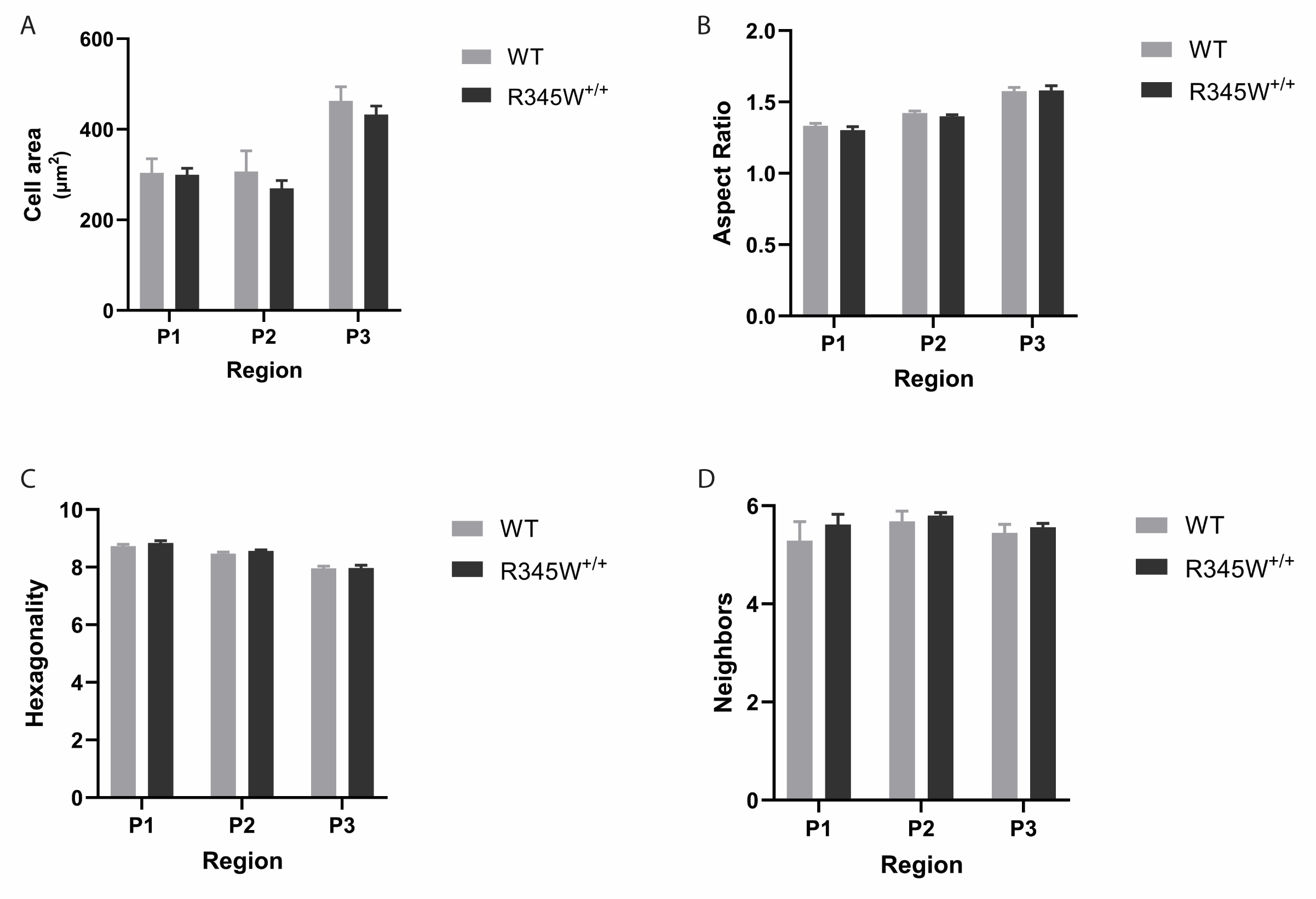

Supplement: Supplementary file 2 — Sup Fig 1 [file 41419_2025_8104_MOESM2_ESM.tif]

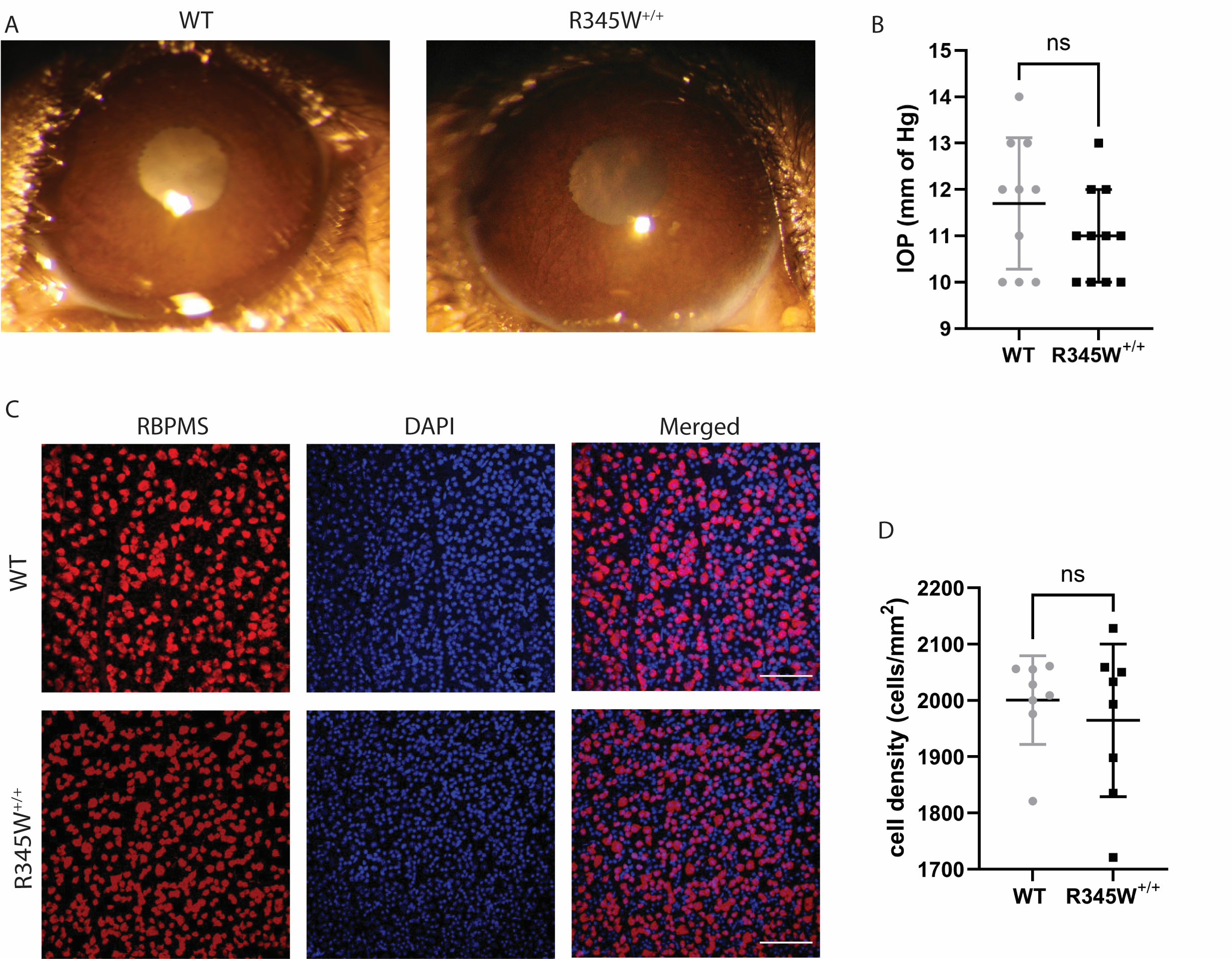

Supplement: Supplementary file 3 — Sup Fig 2 [file 41419_2025_8104_MOESM3_ESM.tif]

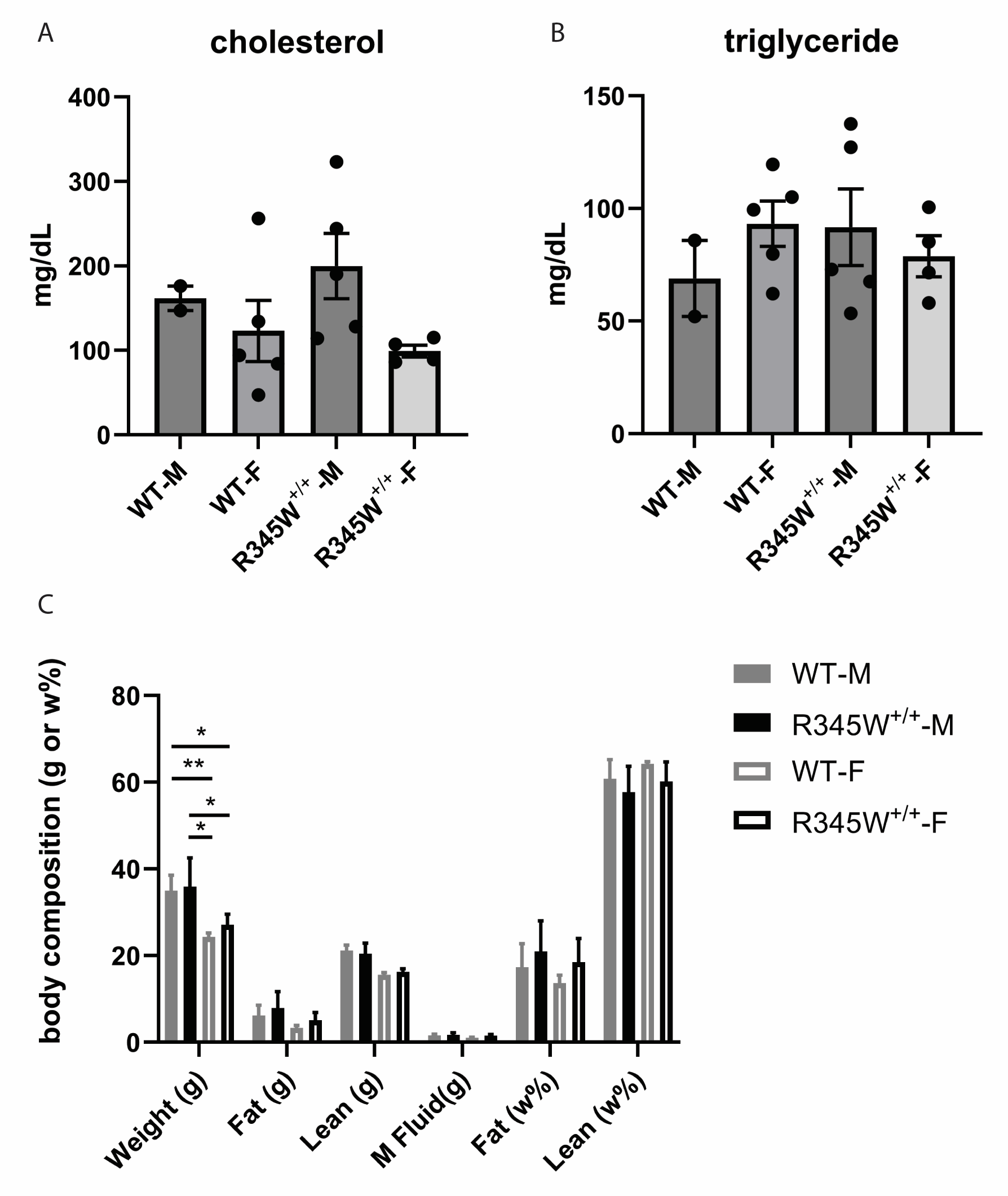

Supplement: Supplementary file 4 — Sup Fig 3 [file 41419_2025_8104_MOESM4_ESM.tif]

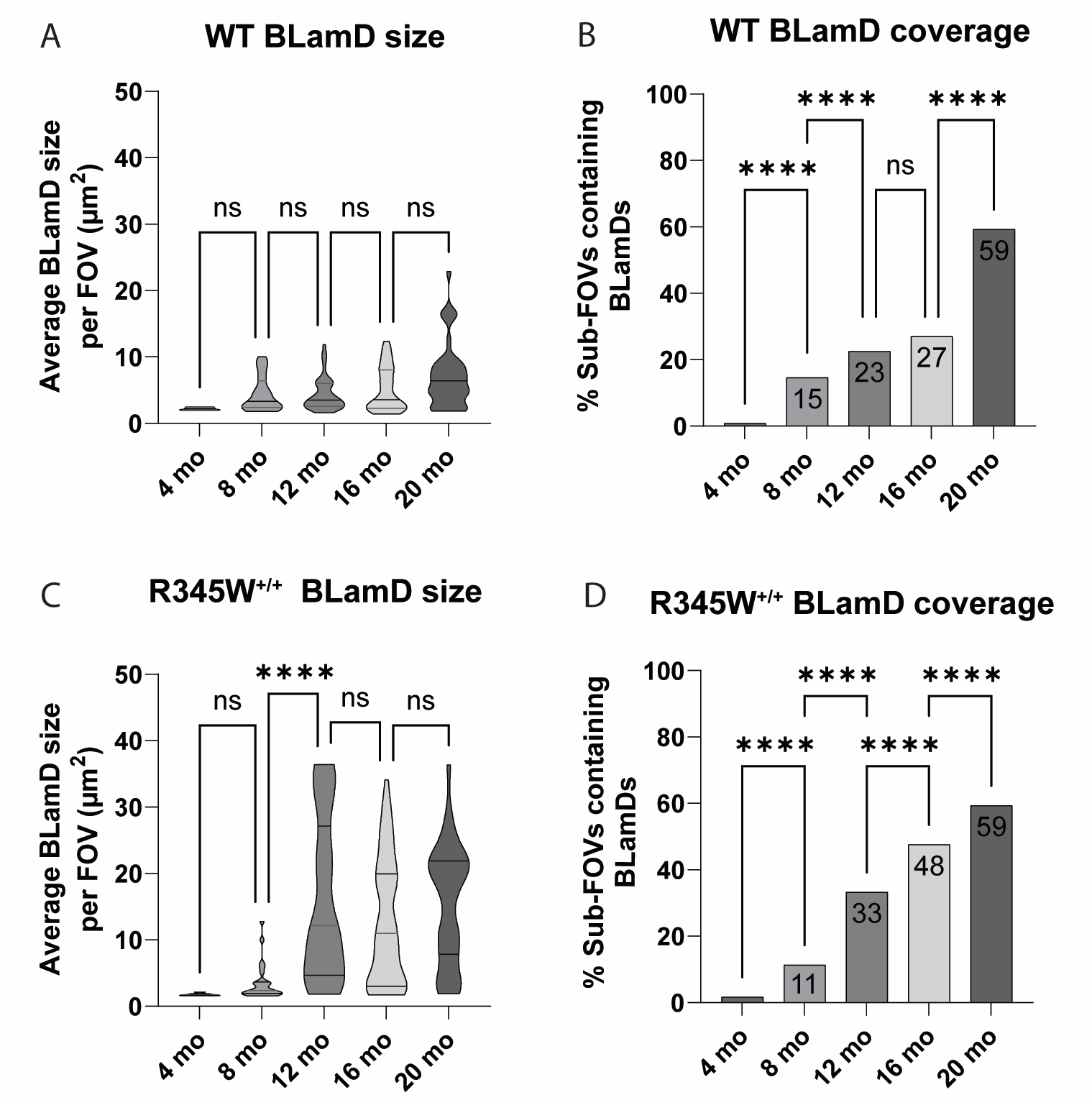

Supplement: Supplementary file 5 — Sup Fig 4 [file 41419_2025_8104_MOESM5_ESM.tif]

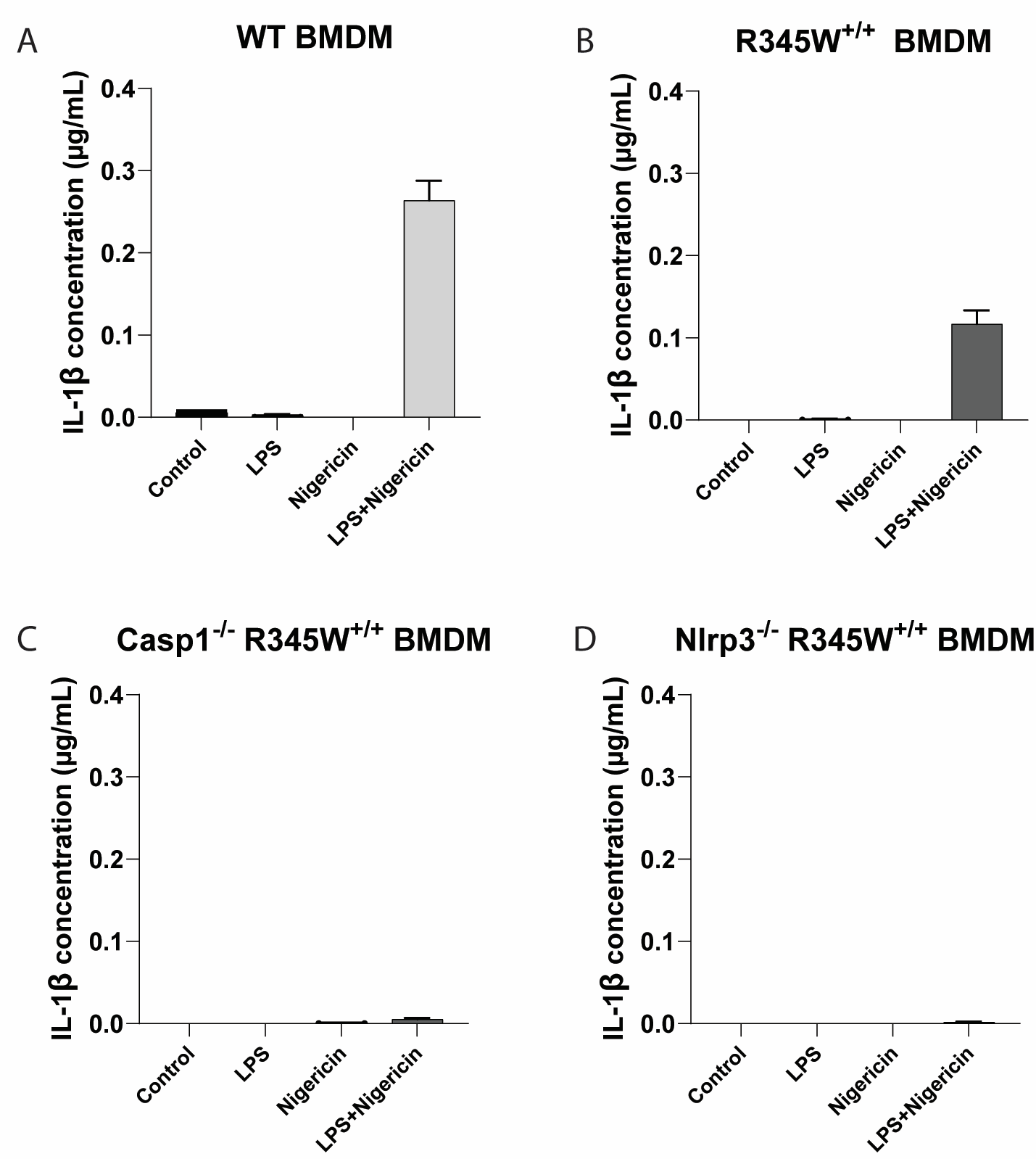

Supplement: Supplementary file 6 — Sup Fig 5 [file 41419_2025_8104_MOESM6_ESM.tif]

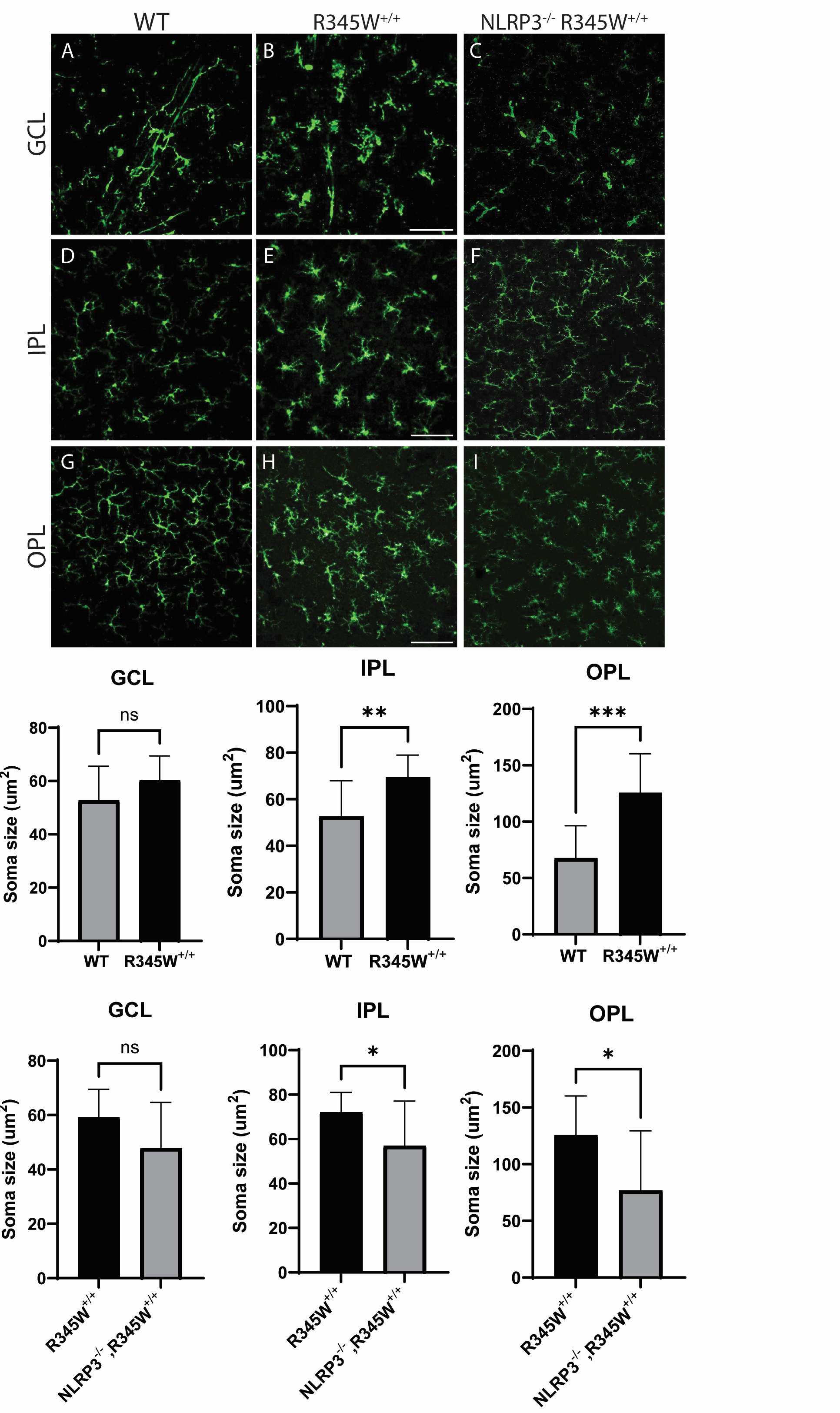

Supplement: Supplementary file 7 — Sup Fig 6 [file 41419_2025_8104_MOESM7_ESM.tif]

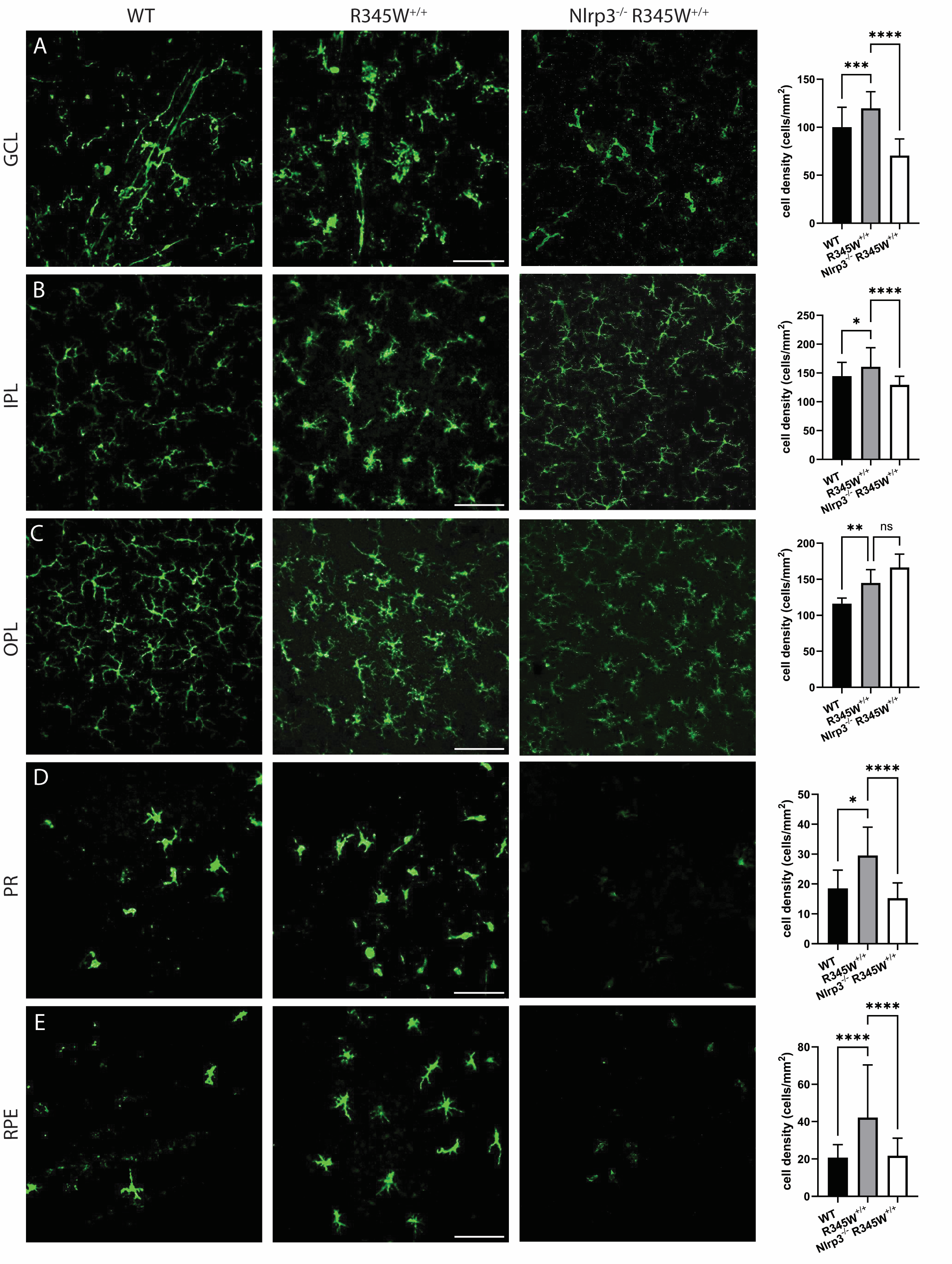

Supplement: Supplementary file 8 — Sup Fig 7 [file 41419_2025_8104_MOESM8_ESM.tif]

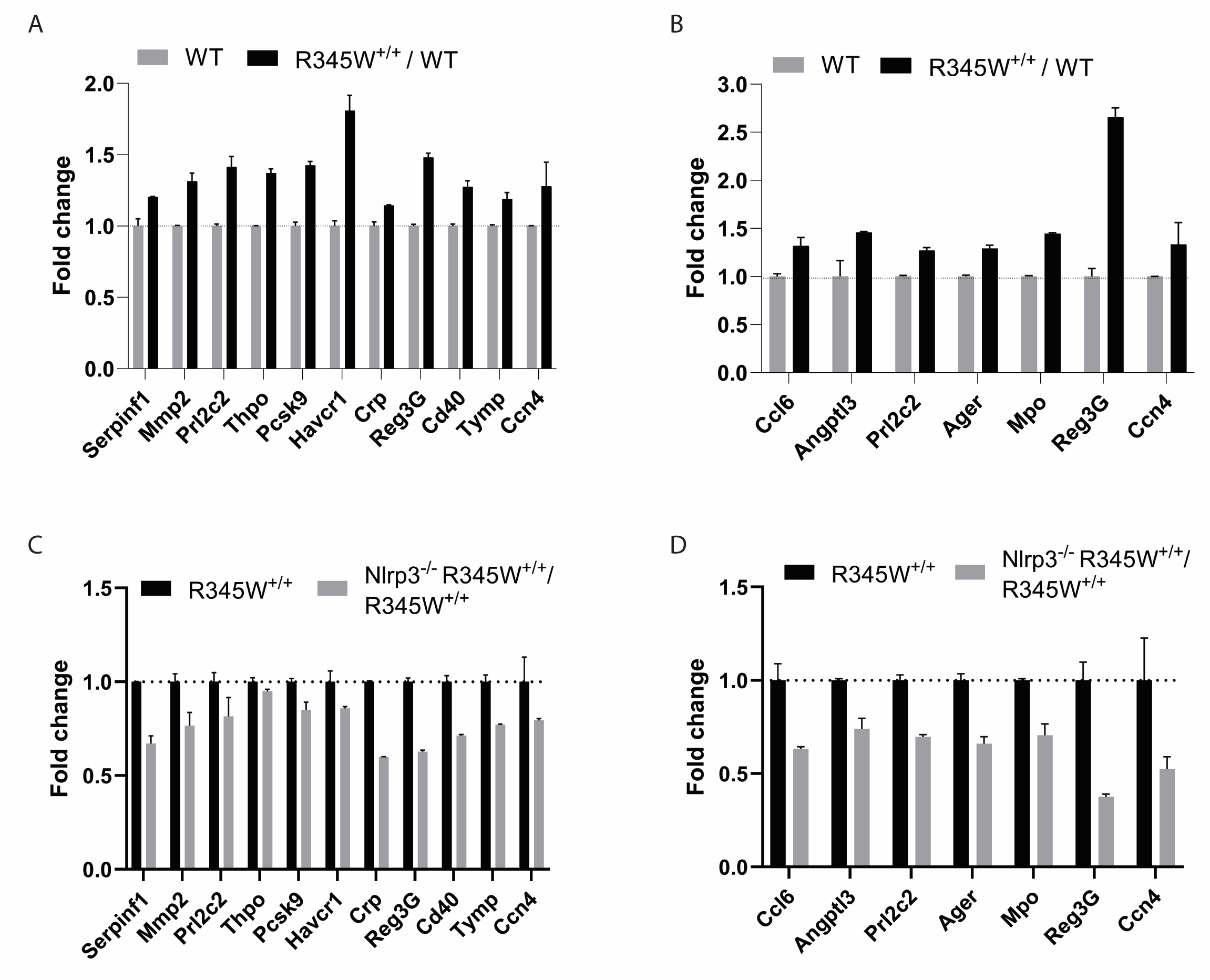

Supplement: Supplementary file 9 — Sup Fig 8 [file 41419_2025_8104_MOESM9_ESM.tif]
